# A Novel Method for Quality Assurance of the Cyberknife Iris Variable Aperture Collimator

**DOI:** 10.7759/cureus.618

**Published:** 2016-05-21

**Authors:** Sarah-Charlotta Heidorn, Nikolaus Kremer, Christoph Fürweger

**Affiliations:** 1 Medical Physicist, European Cyberknife Center Munich; 2 Chief Medical Physicist, European Cyberknife Center Munich

**Keywords:** cyberknife, variable circular aperture collimator, iris, large-area parallel-plate ionisation chamber, quality assurance, field-size determination

## Abstract

Objective: To characterize a novel method for field-size quality assurance of a variable approximately circular aperture collimator by means of dose-area product measurements and to validate its practical use over two years of clinical application.

Methods:  To assess methodical limitations, we analyze measurement errors due to change in linac output, beam tuning, uncertainty in MU delivery, daily factors, inherent uncertainty of the large-area parallel-plate ionisation chamber, and misalignment of the large-area parallel-plate ionisation chamber relative to the primary beam axis. To establish a baseline for quality assurance, the dose-area product is measured with the large-area parallel-plate ionisation chamber for all 12 clinical iris apertures in relation to the 60 mm fixed reference aperture. To evaluate the long-term stability of the Iris collimation system, deviation from baseline data is assessed monthly and compared to a priori derived tolerance levels.

Results: Only chamber misalignment, variation in output, and uncertainty in MU delivery contribute to a combined error that is estimated at 0.2 % of the nominal field size. This is equivalent to a resolution of 0.005 mm for the 5 mm, and 0.012 mm for the 60 mm field. The method offers ease of use, small measurement time commitment, and is independent of most error sources. Over the observed period, the Iris accuray is within the tolerance levels.

Conclusions:  The method is an advantageous alternative to film quality assurance with a high reliability, short measurement time, and superior accuracy in field-size determination.

## Introduction

The CyberKnife (CK) system (Accuray Inc., Sunnyvale, CA) can be equipped with an optional Iris Variable Aperture Collimator (Iris) containing two stacked hexagonal banks of tungsten segments. They together produce a 12-sided aperture with an accuracy ±0.2 mm at nominal distance of 800 mm [1]. The Iris aperture uses multiple aperture sizes and hence benefits improved plan quality and time efficiency [1].

The current manufacturer recommendation (Accuray Physics Essentials Guide 2012, P/N 1023868-ENG A, Accuray Inc. (Sunnyvale, CA)) for quality assurance (QA) suggests monthly film measurements of all 12 field sizes. In order to achieve sufficient accuracy, several hours per measurement series are required [2]. A less time consuming method that achieves the same precision is preferable. The requirements are accurate field size determination, stable and reproducible results, ease of use (clinical utility), and reasonable (small) measurement time commitment. Possible alternatives are scanning water phantom measurements, Iris camera direct imaging [3], Iris beam aperture caliper [4-5], and large-area parallel-plate ionization chamber (LAC) measurements [6]. The LAC is originally intended for proton measurements, and is also proposed to measure dose area product (DAP) in small field energy photon beams [7].

Unfortunately, water phantom measurements are time consuming, and for other suggested methods such as Iris camera direct imaging [3], Iris beam aperture caliper [4-5], and LAC measurements [6], available data is still limited. We present results from  LAC measurements for Iris QA (LAC method), an analysis of limits and influencing factors of the LAC method, and data from the clinical application of the LAC method over 22 months. Further, the long-term Iris performance is investigated and discussed.

## Materials and methods

The Iris collimator contains two stacked hexagonal banks of tungsten segments that together produce a 12-sided aperture that can be continuously varied [1]. The use in the CyberKnife system is restricted to a set of 12 different field sizes (with a diameter d of 5, 7.5, 10, 12.5, 15, 20, 25, 30, 35, 40, 50, and 60 mm specified at a nominal distance of 800 mm). According to the manufacturer, the Iris aperture reproducibility specification is ±0.2 mm at the nominal distance [1].

**Figure 1:**
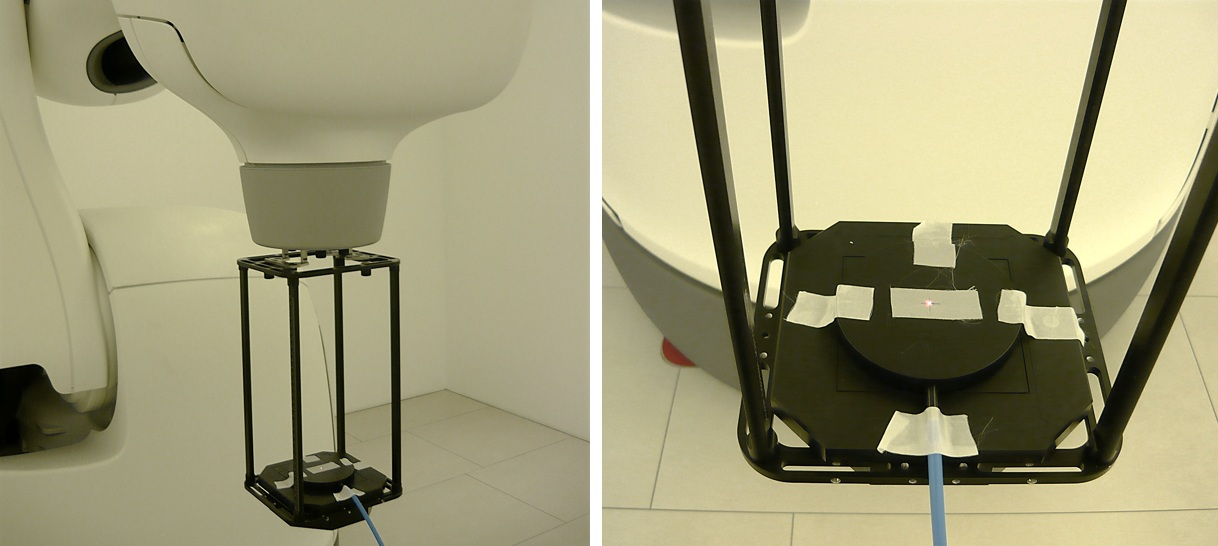
Experimental set-up The LAC is positioned SAD 79.1 cm by means of a hardware accessory and aligned along the central beam axis.

A large-area parallel-plate ionization chamber (TM34070-2,5 Bragg peak chamber, PTW Freiburg, diameter of the active area 81.6 mm, thickness of entrance window 3.47 mm) is placed on top of a hardware accessory that fits into the birdcage assembly (Figure 1). The birdcage is a frame that can be fastened to the collimator assembly where the ionization chamber is arranged at a reproducible position along the central beam axis (SAD 79.1 cm).

For 100 MU each, the uncorrected readings of the 12 Iris apertures dose area products DAP_Iris_(d) and the fixed 60 mm aperture DAP_Fixed_(60mm) are measured three to five times (Unidos Webline, PTW, 10021). The arithmetic mean values for both DAP_Iris_(d) and DAP_Fixed_(60mm) are calculated, and its quotient is determined:

$$\theta (d) = \frac{DAP_{Iris}(d)}{DAP_{Fixed}(60mm)}$$

        

In a similar way, the quotient of baseline data θ_baseline_(d), that have been acquired during commissioning, is calculated.

We analyze different error sources. To assess the change in linac output Δ_output_, multiple measurements (60 exposures, 100 MU, 60 mm fixed collimator) are acquired over the course of 30 minutes, and the standard deviation is determined.

The dependency of θ(d) on primary beam changes is investigated by deliberate detuning of beam symmetry and homogeneity to a level that is clinically not acceptable (parameters: gun voltage  from 10.90 kV to 11.85 kV, grid bias cuttoff voltage from 167 eV to 164 eV). This change corresponds to the worst case scenario encountered in three years of use which does not trip an interlock. The consequence of the detuning is a decrease of the dose in the shoulder area of the profile by approximately 4%. We derive θ_beamchange_(d) and analyze the deviation from baseline results by:

$$\Delta_{beam change}(d) = (\frac{\theta_{beam change}(d)}{\theta_{unmodified}(d)} - 1) * 100$$

We investigate the impact of misalignments during experimental setup. Such a misalignment is possible when exchanging the collimator head from fixed to Iris (or vice versa) because the birdcage and LAC must be removed from the Linac head for exchange. We analyze the influence of different misalignments and check for size dependence. The influence of the positioning of the LAC on θ(d) is derived by misaligning the LAC relative to the central axis (2 mm, 5 mm and 10 mm). The analysis of Δ_misalign_(d) is in analogy to the previous equation:

$$\Delta_{misalign}(d) = (\frac{\theta _{misalign}(d)}{\theta _{aligned}(d)} - 1) * 100$$

For QA, θ(d) is compared to θ_baseline_(d) and given as its percentage deviation via:

$$\delta (d) = (\frac{\theta (d)}{\theta _{baseline}(d)} - 1) * 100$$

In order to define action levels for QA, the Iris aperture reproducibility specification of ±0.2 mm is converted into percentage difference limits of δ(d). The maximal percentage deviations δ_±__0.2_(d) that are within the specifications are the field-size dependent positive and negative limits, respectively. The limits δ_±0.2_(d) are both calculated and measured.

For the measurement, the field size is changed by ±0.2 mm three to five times for all 12 apertures each, 100 MUs are irradiated, θ_measurement, ±0,2mm_(d) is measured, and the arithmetic mean value is calculated. In analogy to equation (2), the limit δ_measurement_,_±0.2_(d) is:

$$\delta _{measurement, \pm0.2}(d) = (\frac{\theta _{measurement ,\pm0.2mm}(d)}{\theta _{baseline}(d)} - 1) * 100$$

**Figure 2:**
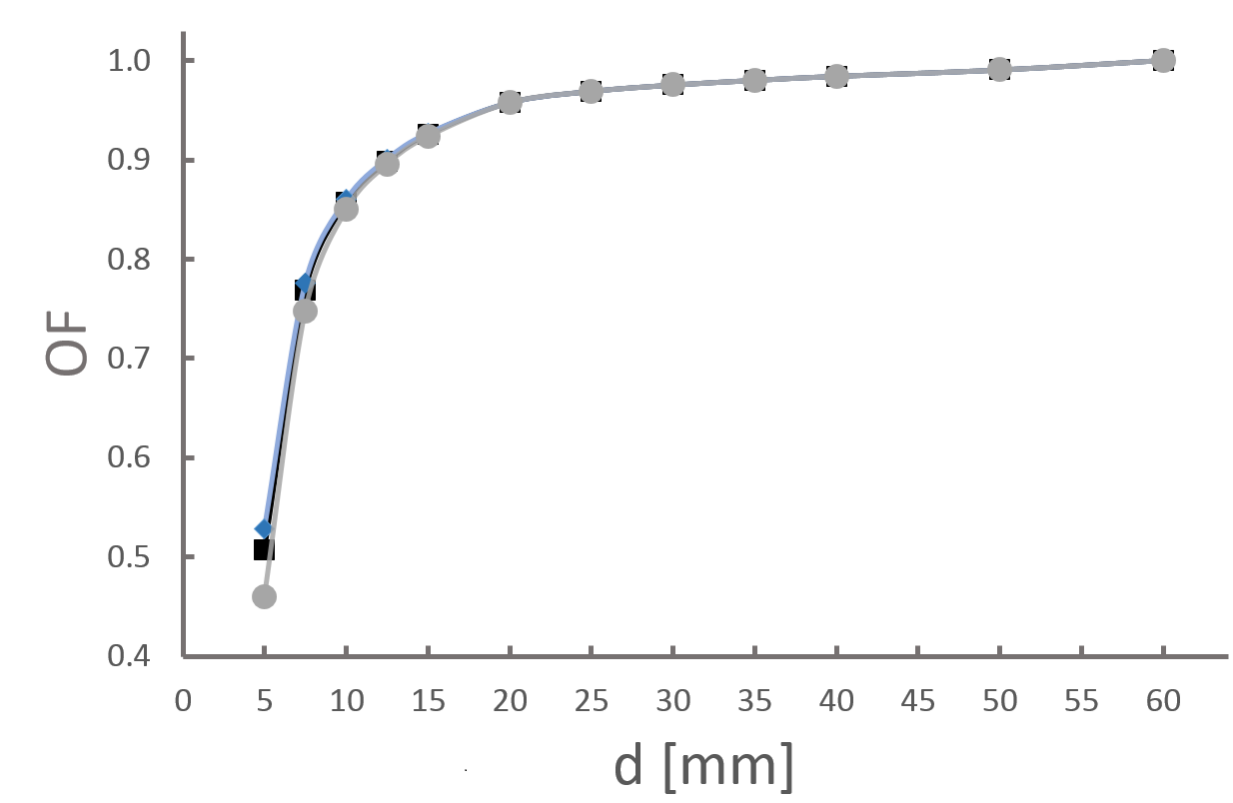
Output factors Measured factors OF (black square) and  calculated factors OF’ (grey circle: -0.2 mm; blue diamond: +0.2 mm) with respect to Iris aperture size.

In an analytical approximation and in analogy to equation (5), the limits δ_c__alculation,__±0.2_(d) are derived from water tank commissioning data by calculating the dose-area product (obtained by radial integration of off-center ratios (OCR) over the chamber area) weighted with the output factor (OF) (Figure 2):

$$\theta _{calculation,baseline} = OF * \int_{0}^{r_{max}} OCR (r,d)2\pi r dr$$

with r_max_  the radius of the LAC sensitive area.

This is compared to the DAP calculated for altered beam profiles θ(r ±2) with a modified radius of ±0.2 mm for the nominal field size and a corrected output factor (OF’) (Figure 2):

$$\theta _{calculation,+0.2mm} = OF' * \int_{0}^{r_{max}} OCR (r,d)2\pi r dr$$

The corrected output factors (OF’) are derived analytically by interpolation between adjacent OFs measured during commissioning.

## Results

### Error analysis

**Figure 3:**
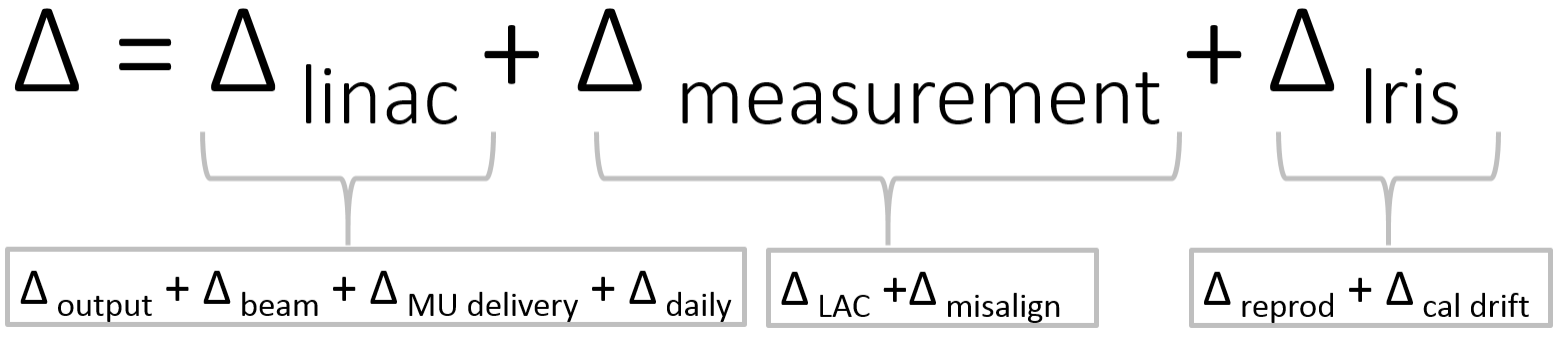
Overview of error sources

An error analysis is performed to validate the LAC method. Different errors may contribute to the quotient θ(d). They can originate from intrinsic linac and Iris characteristics, and the measurement technique (Figure 3). Linac-specific errors Δ_linac_ may arise from daily factors, changes in linac output, primary beam changes, and the uncertainty in MU delivery. Measurement-specific errors Δ_measurement_ can originate from the measurement setup and the inherent uncertainty of the LAC. Iris specific errors Δ_Iris _may consist of the Iris reproducibility and calibration drift over time. Iris specific characteristics are covered in a separate section, and we now analyze linac- and measurement-specific errors. Therefore, we use fixed collimators to exclude the inherent accuracy of the Iris collimator. 

First, linac-specific errors are investigated. Daily factors like temperature, air-pressure, and dose per MU can be neglected. But, the error Δ_output _originating from output changes over the course of a measurement series influences DAP and must be taken into account. Sixty consecutive measurements over the course of 30 minutes show that the error Δoutput is 0.04%.

To investigate the impact of changes in the primary beam, we measure DAP with a detuned primary-beam profile, and calculate θ(d) and the deviation to data from an unmodified beam profile. As a result, DAP, its standard deviation σ_DAP_, and the quotient θ(d) derived for both the detuned and normal beam profile agree within the error. Since we used a beam status that corresponds to the worst case encountered since installation, this is an indication that typical beam changes have no effect on measurements with the LAC method. To estimate the error resulting from the uncertainty in MU delivery (i.e. the output variation when requesting 100 MU), we calculate the mean value of σ_DAP_ in percent for 31 measurement series obtained in 22 months for both a 12.5 mm and a 60 mm fixed collimator. With very similar values of 0.046 ±0.025% (60 mm) and 0.036 ±0.020% (12.5 mm), it is size-independent, and the overall error Δ_output _of DAP due to the MU uncertainty can be estimated as 0.04%.  

Next, measurement-specific errors are derived. The relative error Δ_LAC_ from measurements with the LAC is negligible. We investigate the impact on θ(d) originating from the setup error Δ_misalign_ due to a change in position of the LAC with respect to the central beam axis (misalignment) in the measurement setup. The error Δ_misalign_ is determined for both a small (12.5 mm) and a large (60 mm) fixed collimator. Table 1 shows the mean value of three measurements of θ(d) with a LAC aligned along the central beam axis and of θ_misalign_(d) where the LAC is misaligned by 2 mm, 5 mm, and 10 mm with respect to the central axis.

**Table 1:** Misalignment Deviation between measurements of θ(d) with a LAC aligned along the central beam axis and of θ_misalign_(d) where the LAC is misaligned by 2 mm, 5 mm, and 10 mm with respect to the central axis.

Deviation (in mm)	Δ_misalign_ (60 mm)	Δ_misalign_ (12.5 mm)
2	0.02 ±0.08%	0.10 ±0.02%
5	0.21 ±0.08%	0.19 ±0.06%
10	1.26 ±0.08%	0.41 ±0.04%

The discrepancy is 0.02 ±0.08% (60 mm) and 0.10 ±0.02% (12.5 mm) for a misalignment of 2 mm. A misalignment of 5 mm results in a deviation of 0.21 ±0.08% (60 mm) and 0.19 ±0.06% (12.5 mm), respectively. For a 2 mm and a 5 mm shift, the error Δ_misalgn_ is size-independent. A shift of 10 mm results in a deviation of 1.26 ±0.08% (60 mm) and 0.41 ±0.04% (12.5 mm), and thus size-dependent. For the error estimation, we assumed a misalignment of 2 mm. To conclude, the combined linac- and measurement-specific errors that contribute to θ(d) are approximately 0.2%.

### Characterization of DAPIris(d) and the quotient θ(d) with the LAC method

**Figure 4:**
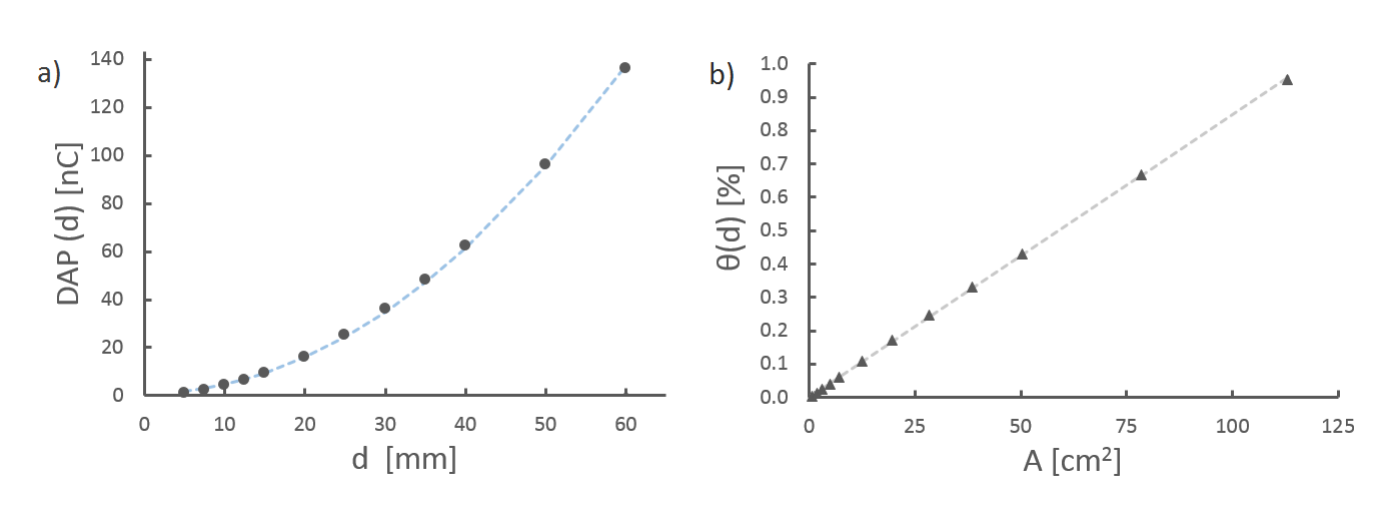
Characterization Relationship between DAP and Iris aperture radius (a) and quotient θ(d) and Iris aperture area (b). The blue dashed line is a fit of the form y = b*x^c^ with an exponent c = 1.935 ±0.004 and b = 0.0493 (a), the grey dashed line is a linear fit y = b*x with b = 0.00846 (b).

We characterize the relationship for one measurement series between Iris aperture and DAP_Iris_(d) (Figure 4a) and its associated quotient θ(d) (Figure 4b). The arithmetic mean values of DAP_Iris_(d) are 2.41 ±0.013 nC, 35.88 ±0.006 nC, and 136.33 ±0.058 nC for Iris apertures of 7.5 mm, 30 mm, and 60 mm, respectively (Figure 4a). The fit is parabolic with an exponent of 1.935 ±0.004 (blue dotted line in Figure 4a) as expected due to the circular surface area of the LAC´s sensitive volume. The arithmetic mean values of the appropriate quotients θ(d) are 0.0168 ±0.00010 at an area of 1.77 cm² (7.5 mm), 0.2503 ±0.0007 at 28.27 cm² (30 mm), and 95.09 ±0.00079 at 113.1 cm² (60 mm) (Figure 4b). As expected, the relationship between the aperture area and the quotient θ(d) is linear (Figure 4b).

### Specification limits

**Figure 5:**
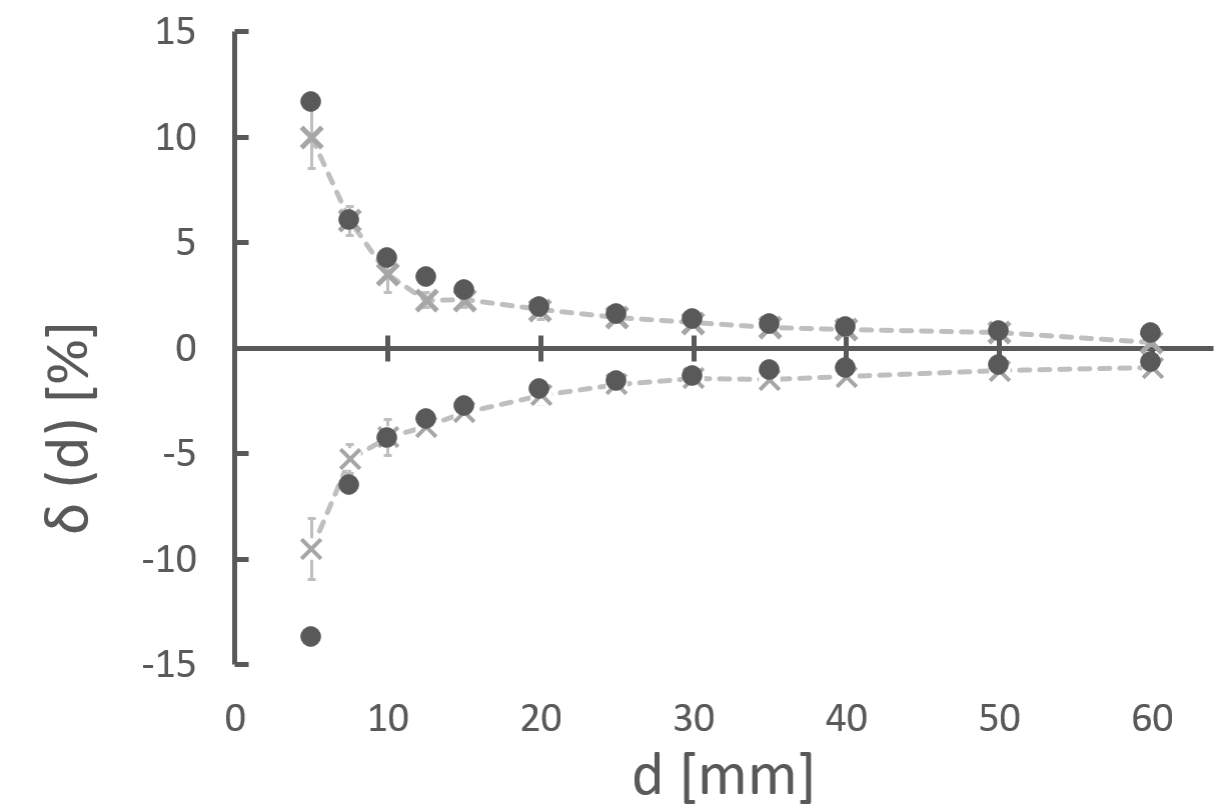
Specification limits Measured (LAC method, grey dotted lines and crosses) and calculated (black circles) specification limits as a percentage deviation from baseline data (for details about δ(d), see methods section).

Figure 5 shows the measured (LAC method, grey dotted lines and crosses) and calculated (black circles) specification limits as a percentage deviation from baseline data (for details about δ(d), see methods section).The measured specification limits are +10.00 ±1.47% and -9.51 ±1.47% (5 mm), 1.21 ±0.211% (30 mm) and -1.41 ± 0.211% (60 mm). The calculated specification limits are, e.g., +11.67% and -13.72% (5 mm), +1.33 and -1.34 (30 mm), and ±0.67 (60 mm).

### Iris characteristics: reproducibility and stability

**Figure 6:**
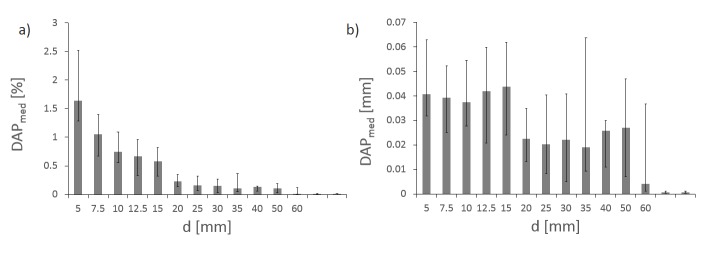
Reproducibility Median standard deviation of 31 DAP measurements for all 12 iris apertures over 22 months in percent (a) and in mm (b). Error bars are first and third quartiles. For comparison, values for fixed 12.5 mm and 60 mm aperture are shown (right side of the x-axis).

In this section, we account for the combined linac- and method-specific errors estimated in the first section. To derive the reproducibility of the Iris, we calculate DAP’s median standard deviation DAP_med_ for 31 measurements (Figure 6a). It decreases with aperture size, from 1.64% for a 5 mm aperture to 0.01% for a 60 mm aperture (Figure 6a). Calculating the absolute reproducibility in millimeters (Figure 6b), we find that the reproducibility for all 12 Iris apertures is equal within the error. The overall Iris reproducibility is below 0.05 mm. In comparison, median standard deviations for fixed cones are minimal because there is no change in field size.

**Figure 7:**
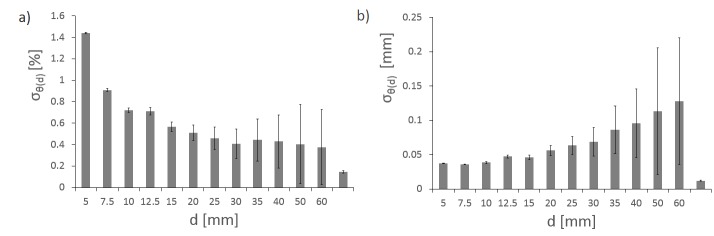
Stability Mean value of standard deviation of the quotient θ(d) from 31 DAP measurements for all 12 Iris apertures over 22 months in percent (a) and in mm (b). For comparison, values for fixed 12.5 mm aperture is shown (right side of the x-axis).

The calibration drift over time is derived by investigating the quotient θ(d) for 31 consecutive QA measurements over a period of 22 months. There is no trend in time recognizable (not shown). When pooling all 31 datasets, the mean value of the standard deviation of the quotient θ(d) is between 1.5% (5 mm) and 0.6% (60 mm), with larger values for smaller Iris apertures (Figure 7a). Translated to absolute variation of the beam diameter (Figure 7b), this corresponds to 0.037 mm (5 mm) and 0.13 mm (60 mm).

### Long-term QA

**Figure 8:**
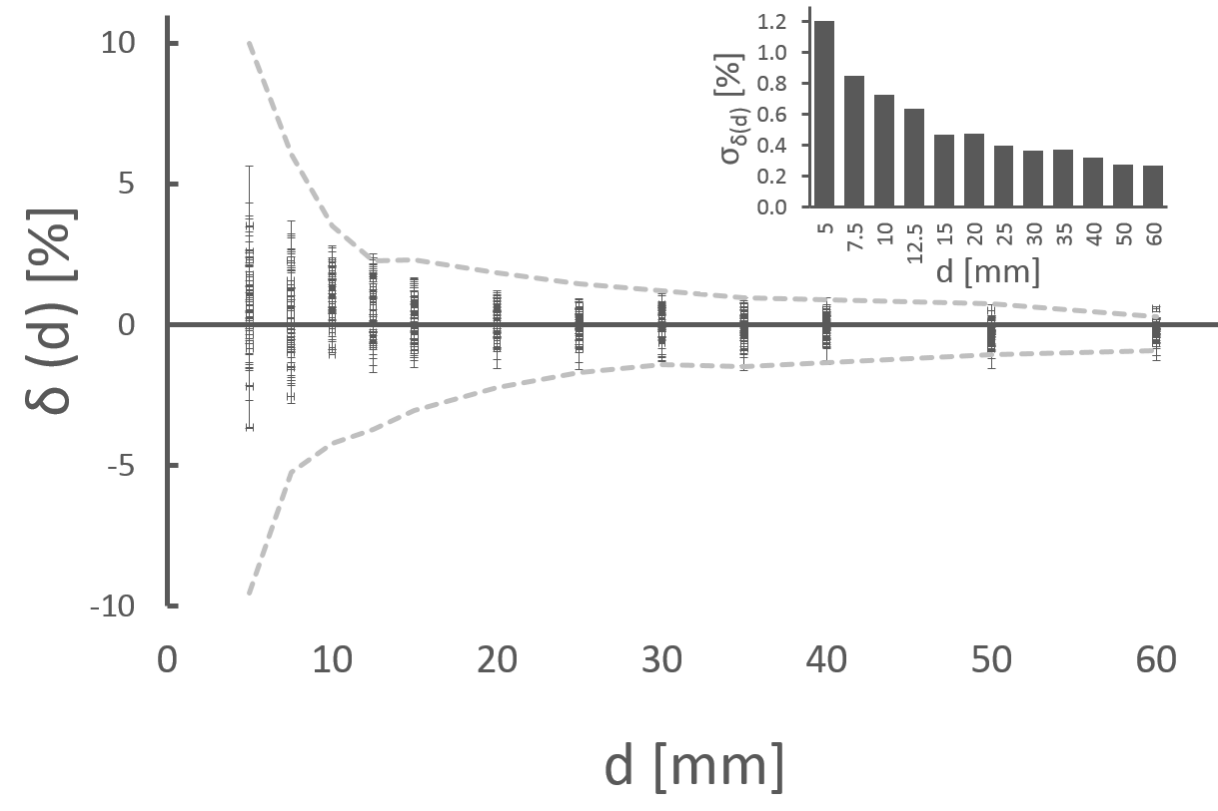
Long-term QA measurements QA measurements for all 12 Iris apertures over 22 months (grey dashed lines: measured tolerace limits, inlet standard deviation of δ(d)).

To interpret the same dataset in terms of clinical acceptability of the Iris collimator, the deviation δ(d) to baseline data is analyzed (black dots in Figure 8). For all 12 apertures, the deviations δ(d) are well within the specification (measured specification, grey dotted lines in Figure 8). The standard deviation of δ(d) from all measurements (inlet in Figure 8) is between 1.2% (5 mm) and 0.27% (60 mm).

The error for the worst case measurement series is 3.63 ±0.63% for a 5 mm collimator. This corresponds to a geometric difference of 0.090 ±0.002 mm. Larger apertures of 20 mm and 60 mm have an error (worst case measurement series) of -0.82 ±0.56% and 0.63 ±0.41%, respectively. This is equal to a geometric discrepancy of -0.082 ±0.006 mm and 0.189 ±0.057 mm.

## Discussion

To evaluate the LAC method, we discuss its accuracy in field size determination, the value of the method for stability and reproducibility, and its clinical utility including expenditure of time.

A linear (parabolic) relationship is expected between θ(d) and Iris aperture area (size), which is confirmed by our data. The residual deviation from linearity (parabolic form) may have its origin in various factors, e.g., the different measurement depths of OF and DAP, backscatter from the plastic support on the birdcage assembly, and the deviation of the real Iris aperture from an ideal radial aperture that is assumed for calculation.

Various factors influence the accuracy of the LAC method. Main contributions come from changes in linac output, the uncertainty in MU delivery, and a misalignment in the setup. The influence from a modification of the primary beam can be neglected, and the LAC method is insensitive to primary beam changes. The error Δ_misalign_ is size-dependent for a 10 mm shift (Table 1, lower row). The reason is that a 10 mm shift moves the penumbra of the 60 mm field very close to the edge of the sensitive volume, which causes a larger difference in the chamber reading. As a conclusion, Δ_misalign _is size-dependent for large misalignments. It is advisable to minimize any misalignments and achieve a precision in every setup below 2 mm.

The validation of a measurement series is done by comparison to baseline data and calculating the derivation δ(d). It is important to keep in mind that baseline data represent a snapshot in time at commissioning. Errors like misalignment, change in output, and uncertainty in MU delivery also will contribute to baseline data. Within this limitation, tolerance values (action levels for QA) in line with Iris technical specifications are established by means of analytical calculation and measurements. Both approaches are in good agreement. Small differences are found for small collimators of 5 mm, 7.5 mm, and 10 mm. This is due to the fact that the OFs are not measured but calculated by interpolation between adjacent Iris apertures sizes. For smaller collimators this has a larger effect because of the increasing gradient of the OF function (Figure 2).

In measurements with the LAC, the Iris collimator displays stable performance, with Iris aperture sizes well within the tolerance limits and high stability over 22 months. Noteworthy, especially small apertures (5 mm, 7.5 mm, and 10 mm) have a much higher precision/repeatability than indicated by the manufacturer. Regarding the clinical use of the smallest apertures, one must take into account two other Iris characteristics beyond basic field size QA: first, the same absolute deviation in aperture size means a higher uncertainty in total dose to the target or patient, which is better represented by percentage deviations in our measurement series, e.g., 0.1 mm corresponds to 0.34% for a 60 mm field but 2.7% for a 7.5 mm field, second, the treatment planning system assumes circular fields and for small collimators, the deviation between the circular field and the real 12-sided field is larger [1]. Keeping all these arguments in mind, small Iris collimators can be clinically used in a moderate and adequate way.

**Figure 9:**
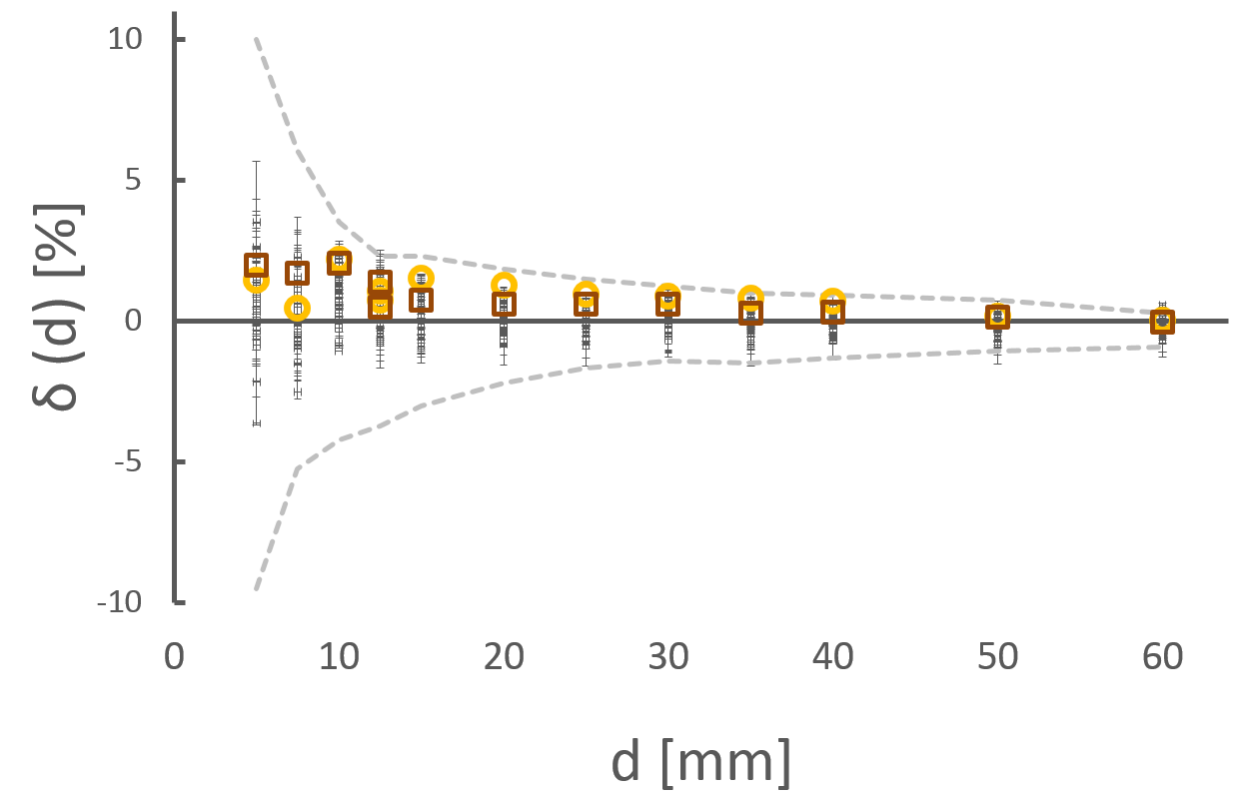
Comparison Misalignment (10 mm, red open squares), beam tune (yellow open circles), and long-term QA measurements (black dots). The grey dashed lines are the measured tolerance levels.

As a visual summary of both the QA data and the impact of key uncertainty factors, Figure 9 compares data acquired with methodical errors to our QA results (red open squares: misalignment of 10 mm; yellow open circles: detuned beam), measured tolerance levels (grey dashed lines), and the QA data aquired during 22 months (black dots). All modified data are within tolerance levels and agree with maximal deviations of the long-term QA. In this manner, the LAC method is demonstrated to be robust against minor errors of the operator and important technical disturbances.

Both setup and measurement with the LAC are straightforward and take less than an hour; so the method can easily be implemented in clinical daily life. The informative value is high because several measurement values are obtained per aperture size, and a mean value is calculated. As a comparison, the film-based standard technique takes several hours, and only one film measurement per aperture size is acquired. Due to these characteristics, the LAC can be considered superior.

## Conclusions

To conclude, the LAC method is capable for accurate determination of field size changes by measuring DAP and comparing with reference data acquired at time of commissioning.  Characteristics of the LAC method are stable and reproducible results, ease of use, and reasonable measurement time commitment of less than one hour. The methodical error is as low as 0.2%. Major error contributions originate from a variation in linac output, uncertainty in MU delivery, and misalignment of the LAC relative to the primary beam axis. As a further result, the Iris has a high reproducibility with a reliable and stable functionality over 22 months.
